# Enhancing the Accuracy of Lymph-Node-Metastasis Prediction in Gynecologic Malignancies Using Multimodal Federated Learning: Integrating CT, MRI, and PET/CT

**DOI:** 10.3390/cancers15215281

**Published:** 2023-11-03

**Authors:** Zhijun Hu, Ling Ma, Yue Ding, Xuanxuan Zhao, Xiaohua Shi, Hongtao Lu, Kaijiang Liu

**Affiliations:** 1 Department of Gynecologic Oncology, Renji Hospital, School of Medicine, Shanghai Jiao Tong University, Shanghai 200001, China; huzhijunjun@outlook.com (Z.H.); zhaox_x@126.com (X.Z.); 2 Library, Shanghai Jiao Tong University, Shanghai 200240, China; maling920827@sjtu.edu.cn (L.M.); xhshi@sjtu.edu.cn (X.S.); 3 School of Electronic Information and Electrical Engineering, Shanghai Jiao Tong University, Shanghai 200240, China; dingyue@sjtu.edu.cn

**Keywords:** gynecological malignancies, lymph node metastasis, multimodal federated learning, multilayer perceptron, convolutional neural network

## Abstract

**Simple Summary:**

Lymph node metastasis is a crucial factor in determining the treatment and prognosis of patients with gynecologic malignancies. Traditionally, medical imaging, including CT, MRI, and PET-CT, are used to detect these metastases. This research introduces a novel approach called “multimodal federated learning” that combines information from these imaging methods to improve the accuracy of lymph-node-metastasis prediction. In simpler terms, this research merges the strengths of multiple-imaging techniques, using advanced computer algorithms to provide a clearer picture of cancer spread. This merger of techniques can provide a more precise diagnosis, facilitate the accurate formulation of treatment plans for patients, and pave the way for similar improvements in other areas of medical imaging.

**Abstract:**

Gynecological malignancies, particularly lymph node metastasis, have presented a diagnostic challenge, even with traditional imaging techniques such as CT, MRI, and PET/CT. This study was conceived to explore and, subsequently, to bridge this diagnostic gap through a more holistic and innovative approach. By developing a comprehensive framework that integrates both non-image data and detailed MRI image analyses, this study harnessed the capabilities of a multimodal federated-learning model. Employing a composite neural network within a federated-learning environment, this study adeptly merged diverse data sources to enhance prediction accuracy. This was further complemented by a sophisticated deep convolutional neural network with an enhanced U-NET architecture for meticulous MRI image processing. Traditional imaging yielded sensitivities ranging from 32.63% to 57.69%. In contrast, the federated-learning model, without incorporating image data, achieved an impressive sensitivity of approximately 0.9231, which soared to 0.9412 with the integration of MRI data. Such advancements underscore the significant potential of this approach, suggesting that federated learning, especially when combined with MRI assessment data, can revolutionize lymph-node-metastasis detection in gynecological malignancies. This paves the way for more precise patient care, potentially transforming the current diagnostic paradigm and resulting in improved patient outcomes.

## 1. Introduction

Gynecological malignancies, especially cervical carcinoma (CC) and endometrial carcinoma (EC), present formidable challenges in women’s health across the globe. Recent statistics from 2020 reported an alarming surge of over one million new diagnoses, underscoring the criticality of effective interventions [1]. Among the diverse metastatic pathways these malignancies exploit, the lymphatic system stands out, with CC and early-stage EC recording metastatic incidences of 15–20% and 10–17%, respectively [2,3]. Traditionally, lymphadenectomy—the surgical removal of lymph nodes—has been pivotal in treating EC and manageable CC cases. Thus, the precision in determining lymph node metastasis (LNM) became a cornerstone of effective treatment.

In 2018, the landscape of medical diagnostics underwent a pivotal transformation. The International Federation of Gynecology and Obstetrics (FIGO) introduced a nuanced approach to CC staging, underscoring the imperative role of LNM detection [4]. Notably, imaging techniques, such as computed tomography (CT), magnetic resonance imaging (MRI), and fluorine-18-labeled fluoro-2-deoxy-D-glucose positron emission tomography–computed tomography (18F-FDG PET/CT) have dominated LNM identification. However, these methodologies have intrinsic limitations. If LNM is confirmed via CT, MRI, or PET/CT, the patient is typically advised to undergo external beam radiation therapy (EBRT) for CC. The repercussions of EBRT can vary, encompassing conditions ranging from pelvic lymphedema to more severe issues such as symptomatic vaginal stenosis, which can detrimentally impact a patient’s quality of life [5]. This underscores the paramount importance of accurate LNM evaluation. Additionally, prominent European medical consortia have advocated for the primacy of PET/CT in nodal assessments for advanced CC [6]. However, despite their widespread utilization, the diagnostic accuracy of these imaging modalities warrants further refinement.

Amid this landscape, this study embarked on a pioneering journey: introducing a multifaceted evaluation framework driven by multimodal federated learning. At its core, this framework employed a composite neural network model that synergistically integrates image data, harnessing the computational strengths of both the multilayer perceptron (MLP) and the convolutional neural network (CNN) [7,8,9,10]. This approach aimed to offer a more nuanced and accurate prediction of LNM. One of the groundbreaking facets of this method was the deployment of federated learning. In an era in which data privacy is paramount, federated learning facilitates inter-institutional data sharing and computation without compromising patient confidentiality [11].

The findings of this study not only present an avant-garde method for LNM assessment, but also underscore the transformative potential of federated learning in medical-image analytics. As we delve deeper into the dataset’s construction, model design, and training methodologies, this study stands as a testament to the evolution of diagnostic paradigms, promising more precise LNM assessments in gynecological malignancies.

## 2. Related Works

As discussed in this section, the study delved into the multifaceted realm of gynecological malignancies, focusing particularly on the pivotal roles of imaging modalities in the assessment of LNM in CC and EC. The essence of the discussion revolves around the paramount importance of accurate LNM assessment, given the metastatic nature of these cancers.

To gain insights into the diagnostic landscape, previous researchers extensively employed CT and MRI modalities. Historically, these tools, which center on morphological characteristics of lymph nodes, such as size, shape, and border consistency, were the cornerstones of diagnosis. Kim et al. [12] and McMahon et al. [13] elaborated on their significance, even though their diagnostic efficiency, as illustrated by sensitivities captured by Choi et al. [14], Bipat et al. [15], Haldorsen et al. [16], Kim et al. [17], and Sarabhai et al. [18], were often subject to scrutiny. In contrast to these traditional methods, PET/CT, spotlighted by Adam et al. [19], offered a broader panorama by integrating morphological imaging with the capability of detecting metabolic alterations. However, as Kitajima et al. [20] pointed out, even this advanced modality was not immune to challenges, especially where micro-metastases in lymph nodes were concerned.

To enhance diagnostic capabilities, especially in the realm of deep learning, the integration of artificial intelligence (AI) has taken center stage. A significant trend observed in the literature was the application of machine-learning and deep-learning techniques for the diagnosis and prognosis of CC. Researchers Rahimi et al. [21] and Matsuo et al. [22] explored the potential of traditional machine learning algorithms, such as decision trees and random forests. In contrast, studies by Al Mudawi et al. [23] and Zhang et al. [24] showcased the prowess of deep-learning methods. Furthermore, research by Dong et al. [25] attested to the power of data integration, successfully amalgamating medical-imaging data with other clinical data to enhance prognostic evaluations.

Shifting the focus to the surgical aspect, some research, notably by Erdem et al. [26], delved deeply into risk factors related to preoperative and postoperative scenarios in CC. Notably, an innovative approach in Zhang et al. [27] employed a multi-dimensional risk assessment strategy. By integrating diverse biomarkers, including coagulation and immune function indicators, researchers gained a more comprehensive understanding of patients’ conditions, setting a benchmark for future investigations.

In the realm of therapeutic optimization, AI and machine-learning techniques began to shine. Findings by Wang et al. [28] were particularly enlightening, unveiling the potential of AI and reinforcement learning in enhancing radiation-therapy planning. This discovery underscored the vast possibilities in the horizon for therapeutic optimization endeavors.

Especially against the backdrop of pathological imagery, the practicality of medical image analysis was emphasized in recent research. A meticulous evaluation of the Cerviray AI^®^ system by Kim et al. [29] highlighted its efficacy in diagnosing late-stage cervical intraepithelial neoplasia. Additionally, work by Liu et al. [30] showcased the potential of weakly supervised deep learning, particularly in identifying LNM from intricate histopathological slides.

In conclusion, a holistic approach to data integration emerged as a disruptive transformation in the field of CC research. Studies like those of Dong et al. [25] and Zhang et al. [27], emphasizing this paradigm shift, amalgamated diverse data sources into a unified model, demonstrating the heightened accuracy and robustness this integration brings to diagnostic and prognostic models. The focus on integrative data-synthesis methods underscores potential trajectories for future research, heralding the advent of a new era in the domain of CC diagnosis and treatment.

## 3. Dataset

This section is structured in three distinct parts. First, the background and characteristics of the study participants are illuminated. Then, the various clinical and laboratory data points collected during the study are detailed. Finally, the strategies adopted for data partitioning are outlined, ensuring both its effective utility and the stringent preservation of patient privacy.

### 3.1. Study Participants and Criteria

This study was a retrospective case-controlled investigation involving patients with EC or CC who underwent surgery between August 2016 and May 2021 at the Department of Gynecological Oncology at Renji Hospital Affiliated with Shanghai Jiao Tong University School of Medicine. The research involved a total of 567 patients, comprising 423 cases of CC and 144 cases of EC. The median age of the participants was 49 years, ranging from 19 to 78 years. Through meticulous histopathological analysis, LNM was identified in 89 CC patients, accounting for 21.04%, and 14 EC patients, or 9.72%. An additional observation delved into the distribution of lymphvascular space invasion (LVSI) in patients who were diagnosed with CC or EC, as detailed in Table 1.

Eligible patients met the following criteria: (1) they had been diagnosed with CC (2009 FIGO stage IA1 with LVSI to IIA2) or EC; (2) They had undergone LNM assessment using at least one of the following modalities—MRI, CT, or PET/CT; and (3) they had received surgical treatments such as radical hysterectomy, radical trachelectomy, or total hysterectomy with pelvic lymphadenectomy (with or without sentinel lymph nodes, [SLNs) or para-aortic lymphadenectomy, either through laparoscopic or abdominal procedures. Exclusion criteria included the following: (1) those patients who underwent neoadjuvant chemotherapy or preoperative pelvic radiotherapy; (2) individuals who received lymphadenectomy before the main surgical procedure; and (3) patients who did not undergo lymphadenectomy.

The study recorded pertinent clinical details of the patients, which included age, tumor histopathological attributes, results derived from imaging evaluations concerning LNM, and the postoperative histopathological diagnosis of LNM. This study secured approval from the institutional Ethics Review Board of Renji Hospital Affiliated with Shanghai Jiao Tong University School of Medicine (KY2019-154).

### 3.2. Clinical and Laboratory Data

The dataset employed in this research was sourced from the Gynecological Oncology Department of Renji Hospital. It included real patient data from individuals diagnosed with CC and EC and comprised the field information set out in Table 2.

**Figure 1:**
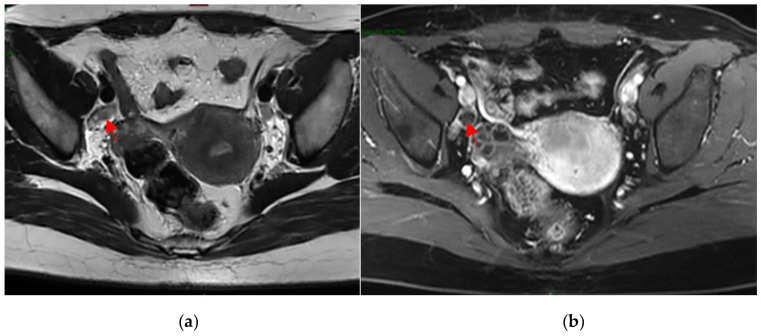
The right external lliac lymph node (arrow) of a 40-year-old EC patient. T2-weighted MRI (**a**) and contrast-enhanced T1-weighted MRI (**b**).

In addition to the above-mentioned fields, the dataset also encompassed image data obtained through preoperative CT and MRI evaluations. Every patient underwent preoperative CT and MRI assessments, resulting in corresponding image data. These images were essential for the analysis and assessment of lymph node metastasis in gynecological malignant tumors. These image data were presented in two-dimensional or three-dimensional forms and included detailed structures and features of the patient’s pelvic and abdominal regions. In the current model, only MRI images were utilized as input for the evaluation of potential lymph node metastasis. Within the images, regions containing possible metastatic lymphatic tissue were segmented and annotated (Figure 1).

### 3.3. Data Partitioning

To ensure patient privacy and to fully utilize the multi-modal data, this study employed an innovative data-management strategy, dividing the dataset into two independent types of clients, Client 0 and Client 1, to simulate a real-world multi-data owner environment. Client 0 encompassed 226 samples, including 111 positive samples and 115 negative samples, while Client 1 consisted of 341 samples, subdivided into 226 positive samples and 115 negative samples. Both data holders combined text and image data, a design that not only safeguarded patient privacy, but also facilitated the optimal integration and utilization of multi-modal data. Additionally, the data within each “Client” category was further segmented into training and testing sets, serving as validation sets for each other, with a ratio of 8:2 between the training and testing sets, thereby achieving a rational distribution and effective validation of the dataset, as shown in Table 3.

## 4. Materials and Methods

In its quest to improve LNM diagnosis in gynecological malignancies, this study crafted an integrated method that blended non-image clinical data and MRI image data within a federated-learning framework. This approach ensured enhanced prediction accuracy, while safeguarding data confidentiality.

The solution hinged on a composite neural network that operated within this federated-learning environment. By adeptly merging diverse data sources, the model delivered more precise predictions for LNM. Whether training on non-image clinical data, MRI image data, or both, the system demonstrated adaptability and efficiency, marking a significant advancement in gynecological malignancy research.

### 4.1. Overall Model Architecture

This study introduced a novel federated-learning framework that operated across various local clients, each of whom was emblematic of distinct medical institutions or data sources. These clients processed two types of multimodal data: non-image clinical data ($X_{text}$) and MRI image data ($X_{image}$). The model was a composite neural network that seamlessly integrated the capabilities of the MLP and the CNN, respectively, to process these data. The fusion of outputs from both MLP and CNN components was achieved through the Softmax activation function, which is formulated as:

(1) $$P(\frac{y}{x})=Softmax(\alpha \cdot f\left(W_{mlp}\cdot X_{text}+b_{mlp}\right)+\beta \cdot f\left(W_{cnn}\cdot X_{image}+b_{cnn}\right))$$

where *α* and *β* are learnable weight parameters that ascertain the significance of each component’s output in the consolidated result. The Softmax function ensured a normalized prediction and was defined by the following equation:

(2) $$S\left(x_{i}\right)=\frac{e^{x_{i}}}{\sum_{j=1}^{N}e^{x_{j}}}$$

where $x_{i}$ represents the *i*th element in the prediction and *N* denotes the total count of the predictions. 

By leveraging the potential of this composite neural network, each local client independently discerned and produced predictive outputs. Instead of sharing the raw data, which may have raised privacy concerns, only the model parameters were transmitted to a central server. This server aggregated the parameters from different clients and undertook global optimization. This methodological approach not only safeguarded data privacy, but also fostered a culture of collaborative knowledge-sharing across institutions. Post-optimization, the central server circulated the refined parameters back to each client, establishing a recursive learning cycle. Through this iterative process, clients collectively learned from each other, enhancing their individual and collective predictive accuracies.

**Figure 2:**
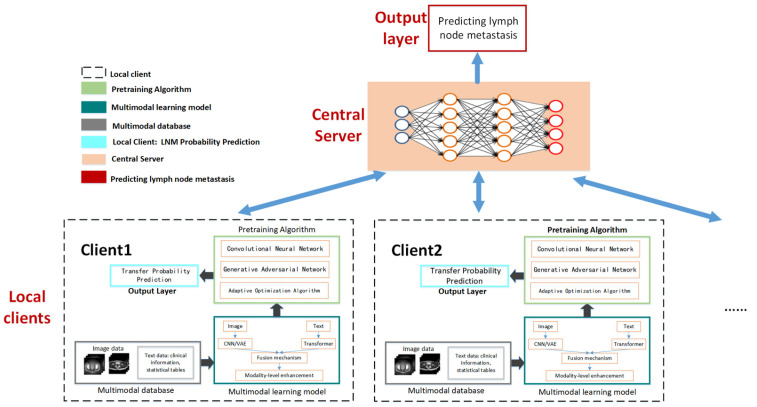
Multimodal federated-learning framework.

By harmoniously amalgamating multiple data sources, utilizing the strengths of both the MLP and the CNN, and adopting federated learning for training, the model furnished a probability metric for LNM. This pioneering approach augmented diagnostic accuracy, endowed flexibility, ensured data privacy, and paved the way for a more nuanced and personalized diagnosis of gynecological malignant tumors, as depicted in Figure 2.

### 4.2. Text Data Model

To enhance the prediction of lymph node metastasis, this study introduced a model tailored for non-image clinical data, which included both pathological findings related to LNM and expert evaluations of imaging techniques such as MRI, CT, and PET/CT. Built upon an MLP framework, this model was adept at capturing key information related to LNM from this clinical dataset.

The MLP was structured with an input layer, several hidden layers, and an output layer. The design ensured the flexibility required to adjust the number of neurons in the hidden layers, a feature that was crucial for discerning the intricate relationships between input features and LNM. The computational representation of the model’s output was determined by the following equation:

(3) $${MLP}_{output}=f(W_{mlp}\cdot X_{text}+b_{mlp})$$

where f is the activation function that transforms the output of the linear function to the non-linear function, allowing the model to learn more intricate data representations. $X_{text}$ is the input data, $W_{mlp}$ represents the weight parameters of the MLP component, and $b_{mlp}$ is the bias term.

This tailored approach not only integrated findings from clinical diagnostic procedures, but also synthesized evaluations from various imaging techniques, thereby amplifying the model’s predictive accuracy for LNM. By converging these diverse data sources, the methodology promised a more holistic and precise prediction, setting a benchmark in the realm of gynecological malignancy research.

### 4.3. MRI Image-Processing Model

Radiological assessments, which are traditionally reliant on the manual expertise of radiologists, bring invaluable insights but also introduce an element of subjectivity, due to human variance. Recognizing this, this study sought to introduce more consistent and objective evaluations by developing an AI model that was tailored for MRI image interpretation, specifically targeting LNM in gynecological malignancies.

To achieve this, this study harnessed a tailored CNN amalgamated with the U-NET architecture for the nuanced interpretation of MRI image data that were pertinent to LNM. This combination ensured both depth in feature extraction and precision in region segmentation.

The CNN structure, designed for MRI images, consisted of multiple convolutional layers, pooling layers, and fully connected layers. These layers worked cohesively to meticulously extract and refine deep features from the MRI images that were related to LNM. The computational output of this network was defined as follows:

(4) $${CNN}_{output}=f(W_{cnn}\cdot X_{image}+b_{cnn})$$

where $X_{image}$ is the MRI image data input, $W_{cnn}$ denotes the weight parameters of the CNN component, $b_{cnn}$ is the bias term, and f again denotes the activation function.

To ensure precise interpretation and the segmentation of MRI image data that were related to LNM, our model integrated a CNN with the U-NET architecture. The U-NET, renowned for its precision in region segmentation, is systematically designed with input blocks, downsampling blocks, upsampling blocks, and a final output block. As MRI T1W sequences are processed, they traverse the U-NET, starting from the input block. The subsequent downsampling phase not only reduces the image’s spatial dimensions, but also amplifies feature channels, thanks to convolutional layers that are paired with Leaky ReLU activation functions. This ensures a detailed extraction of the salient features that are indicative of LNM.

After downsampling, the model underwent the upsampling phase. Here, the original image dimensions were restored, while the depth of the extracted features was maintained. The final step involved the output block, which utilized convolutional layers in tandem with a Softmax activation function. The result was pixel-level classifications that accurately highlighted potential regions of LNM.

Turning our attention to the data underpinning this research, they were rooted in the expertise of two senior radiologists, who reassessed patient MRI scans, providing crucial diagnostic labels. This dataset consisted of MRI T1W sequences, with each sequence containing 1–2 images that were suspected of showing signs of lymph node metastasis. Crucially, these images were annotated by the radiologists, marking the lymph node regions in detail. While the current study prioritized the detection of LNM presence, we recognize that the possible depth and potential of future studies that could delve into the specific characteristics or types of lymph nodes.

Upon the completion of the annotation phase, the MRI image-processing model was deployed, with its primary function being segmentation and feature extraction from the images. These sequence images were then subjected to distributed training across two distinct clients, and further enhanced with cross-validation. The model’s operational flow commenced with image classification, which segued into segmentation using the CNN, culminating in the autonomous identification and annotation of potential LNM regions.

**Figure 3:**
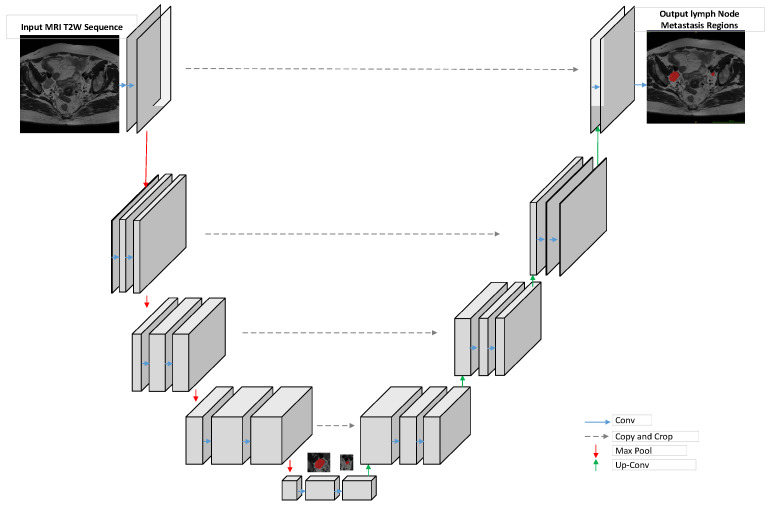
A precise MRI image-processing model for diagnosing lymph node metastasis.

With its intricate segmentation capabilities, the model offered invaluable insights, enabling precise localization of potential anomalies in gynecologic tumor regions. This fusion of CNN and U-NET architectures not only allowed for a comprehensive analysis, but also set a new benchmark for LNM diagnosis in MRI images. Through this integrated approach, the model stood as a formidable tool, heralding a new era in gynecological malignancy research, as shown in Figure 3.

### 4.4. Multimodal Fusion

In the pursuit of refining the diagnosis of LNM in gynecological malignancies, the role of integrative methodologies, particularly the amalgamation of clinical text data and MRI image data, has taken center stage. One significant dimension of this integrative approach was manifest in the “multimodal fusion” model.

The “multimodal fusion” model was established in response to the challenges presented by isolated data sources. It seamlessly combined text-based clinical insights, predominantly drawn from radiologist evaluations, with the diagnostic outputs generated by the advanced MRI image-processing model. By leveraging the precision of AI-driven image analyses and the nuanced insights of experienced radiologists, this fusion strategy aspired to substantially elevate the accuracy of LNM predictions.

Building upon the integrated approach, this section (titled “Multimodal Fusion”) elu-cidates how to harmoniously merge two disparate data sources—text and image—to enhance diagnostic precision. By harnessing the inherent strengths of both data types, the model endeavored to present a comprehensive and nuanced picture, especially con-cerning LNM predictions. The ensuing discussion provides insights into the fusion technique and its implications and details the loss function employed during the model’s training phase.

The fusion result was shown in Equation (1). Through this methodology, it was possible to integrate two types of data (text and image) and to produce a comprehensive prediction regarding LNM for patients. During the model-training phase, the cross-entropy loss function was used to measure the difference between the predicted value and the actual label. It was defined as follows:

(5) $$L=-\sum_{i=1}^{N}y_{i}\log\left(p_{i}\right)$$

where *N* represents the number of samples, $y_{i}$ is the true label, and $p_{i}$ is the prediction probability generated by the model for that sample. This loss function was commonly employed in classification problems, as it directly measured the difference between the model’s predicted probability distribution and the actual label distribution.

### 4.5. Federated-Learning Training

The methodological exposition and underscored federated-learning strategy was underpinned by the federated averaging (FedAvg) [31] algorithm. This innovative approached permitted each participating client, representing distinct medical institutions, to train the model on their local dataset. Optimal data protection and confidentiality were ensured by harnessing the various insights from multiple institutions.

During the training process, each client refined the model parameters based on their local data and, subsequently, transmitted these parameters to a centralized server. This server then undertook the crucial task of averaging the received parameters from all the clients. The formula governing the federated averaging process is expressed as:

(6) $$w=\frac{1}{k}\sum_{k=1}K(\frac{n_{k}}{n})w_{k}$$

where *w* is the model parameter on the server, $n_{k}$ is the number of data in client k, *n* is the total number of data from all clients, and $w_{k}$ is the model parameter uploaded by client k.

After the computation of the averaged parameters, the server updated its global model. This iterative mechanism ensured the model’s continual evolution, incorporating insights from every contributing institution. Through uniform dataset division, cross-validation among clients was further facilitated, thereby enhancing the model’s accuracy.

**Figure 4:**
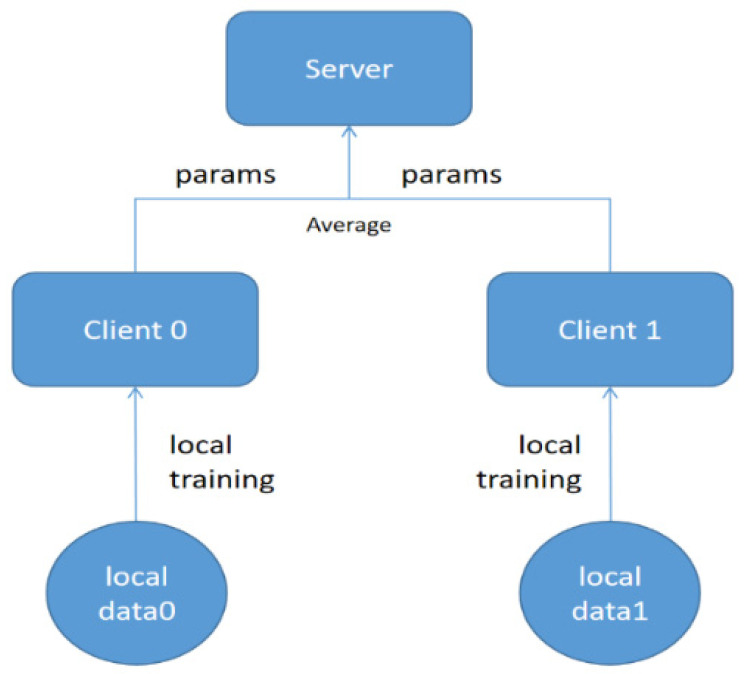
Federated-learning training.

This federated-learning approach, augmented by the FedAvg algorithm, offered a twofold advantage. First, it allowed the model to leverage the expansive and varied data pools of multiple hospitals. Second, it upheld the integrity of data privacy, guaranteeing that raw data remained confined to its origin. Hence, the methodology not only amplified the predictive accuracy for LNM, but also epitomized collaborative, privacy-centric research in medical imaging (Figure 4).

### 4.6. Evaluation Metrics

This research conducted a comparative analysis of the proposed federated-learning model and traditional statistical methods for diagnosing LNM. The conventional statistical analysis was performed using the Statistical Package for the Social Sciences (SPSS) software version 23.0 (International Business Machines Corporation, Armonk, NY, USA), with histopathological data as the primary diagnostic criteria. The SPSS was used to calculate and present the sensitivity, specificity, positive predictive value (PPV), negative predictive value (NPV), and accuracy of CT, MRI, and PET/CT by groups. In contrast, the federated-learning model produced its own set of metrics, including sensitivity, specificity, and accuracy, offering a fresh perspective on LNM prediction. To measure the performance differences, all comparative *p*-values were computed using Pearson’s chi-squared test. Furthermore, the areas under the curve (AUCs) for each group were compared employing the DeLong and DeLong method. In all evaluations, statistical tests were two-sided, and a *p*-value less than 0.05 indicated statistical significance.

This comparative approach emphasized the enhanced capabilities of integrating multimodal data and advanced federated-learning techniques in providing a more comprehensive and precise assessment of LNM risk, compared with traditional methods.

## 5. Results

### 5.1. Evaluating the Efficacy of Individual and Combined Imaging Modalities: CT, MRI, and PET/CT

The participants were divided into seven groups, based on the imaging examinations they underwent preoperatively: CT (439); MRI (440); PET/CT (393); CT and MRI (C-M, 336); CT and PET/CT (C-P, 308); MRI and PET/CT (M-P, 292); and CT, MRI, and PET/CT (C-M-P, 230). The LNM assessment data, stratified according to the histopathology data of each group, are shown in Table 4.

This study embarked on an assessment to compare different imaging modalities in detecting LNM. Specifically, CT results demonstrated a sensitivity of 32.63%, a specificity of 92.15%, and an AUC of 0.624 (0.555–0.693). In comparison, MRI interpretations presented a slightly enhanced sensitivity at 35.9%, a specificity of 93.37%, and an AUC of 0.646 (0.571–0.721). Among these, PET/CT emerged as the most efficient, with a sensitivity of 57.69%, a specificity of 85.71%, and an AUC of 0.717 (0.647–0.787). Notably, PET/CT’s sensitivity and NPV surpassed those of CT and MRI, although the AUC comparisons between them were not statistically significant (*p* = 0.0846).

In exploring the synergistic effects of combined modalities, there were significant differences in sensitivities and specificities, particularly between the C-M and C-P, C-M and M-P, and C-M and C-M-P groupings. Nonetheless, no prominent differences emerged when evaluating other combined examination groups. Moreover, the diagnostic efficacy of PET/CT was accentuated when it was compared to CT and C-P, as well as MRI and M-P, with the AUC values not suggesting any pronounced dominance in LNM detection.

**Figure 5:**
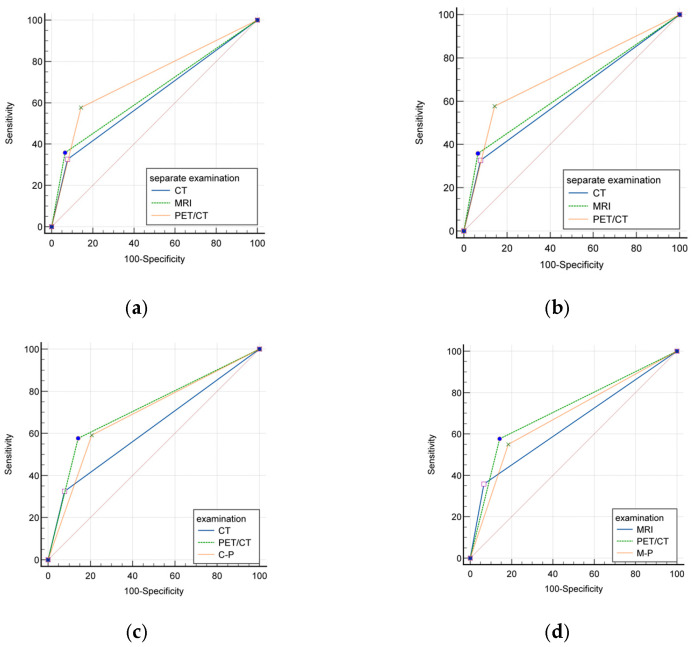
ROC curves for CT, PET/CT, MRI, C-M, C-P, M-P, C-M-P in lymph node assessment. Comparisons of AUCs between the groups of separate examination showed that the AUC of PET/CT was significantly superior to that of CT but there was no significant difference to MRI (*p* = 0.0172, *p* = 0.0846, respectively) (**a**). There was no significant difference of AUC between combined examination groups (*p* > 0.05) (**b**). AUC did not show more obvious superiority in lymph node assessment when CT was compared with C-P and when MRI was compared with M-P, respectively; (*p* > 0.05) (**c**,**d**).

Detailed outcomes of these evaluations are comprehensively tabulated in Table 5 and graphically represented in Figure 5.

### 5.2. Multimodal Federated-Learning Framework Evaluation

**Figure 6:**
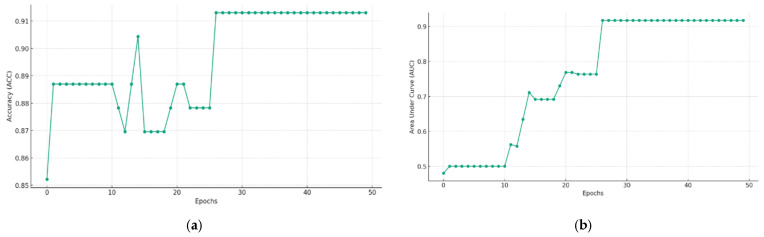

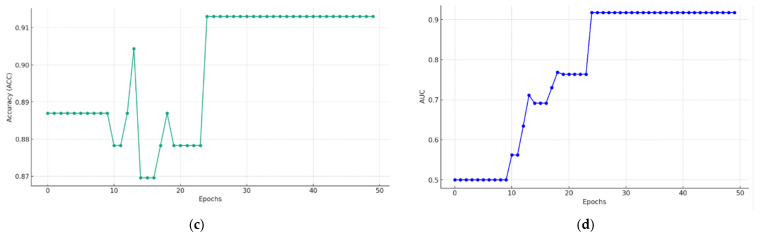
(**a**) Training ACC (accuracy) without MRI image data; (**b**) training AUC without MRI image data; (**c**) training ACC with MRI image data; (**d**) training AUC with MRI image data.

In this study, a multimodal federated-learning framework was employed to assess LNM in 567 samples, of which 226 were used for training, 115 for internal validation, and 115 for external validation. Multiple experiments were conducted on the internal test set to evaluate the model’s performance under two different conditions: (1) without MRI image data, achieving a sensitivity of approximately 92.30%, a specificity of approximately 92.15%, and an accuracy of approximately 92.17%; and (2) with both MRI images and evaluation data, achieving a sensitivity of 94.12%, a specificity of 96.69%, and an accuracy of approximately 95.16% (Figure 6).

**Figure 7:**
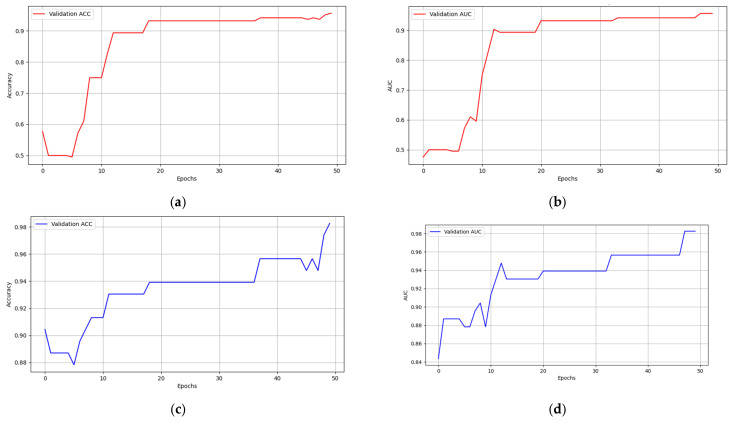
(**a**) Validation ACC (accuracy) without MRI image data; (**b**) validation AUC without MRI image data; (**c**) validation ACC with MRI image data; (**d**) validation AUC with MRI image data.

The experimental results on the validation set were similar. Without MRI image data, the model achieved a sensitivity of 92.31%, a specificity of 93.14%, and an accuracy of 93.04%. However, when combined with MRI imaging and evaluation data, the sensitivity was approximately 92.31%, the specificity was about 98.04%, and the accuracy was around 96.5%, as depicted in Figure 7.

**Figure 8:**
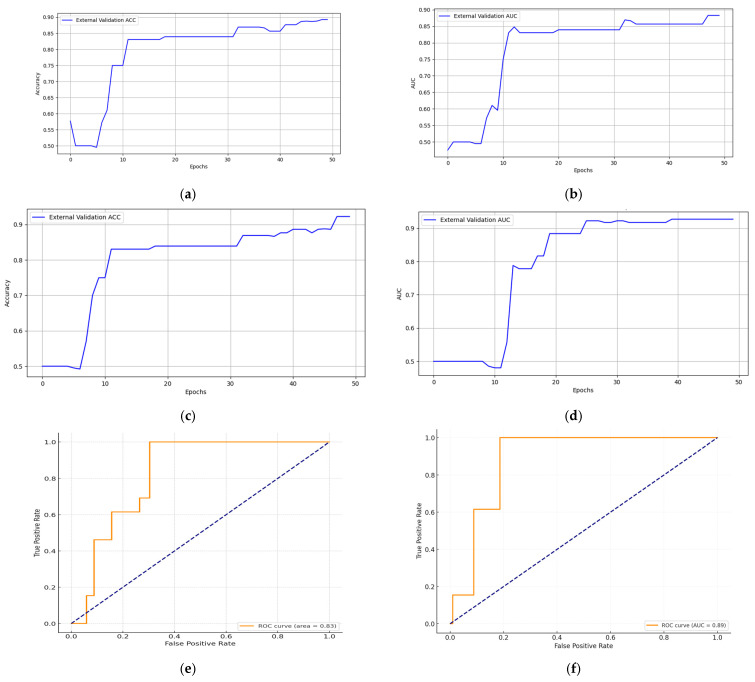
(**a**) External validation ACC (accuracy) without MRI image data; (**b**) external validation AUC without MRI image data; (**c**) external validation ACC with MRI image data; (**d**) external validation AUC with MRI image data; (**e**) external validation ROC curves without MRI image data (AUC = 0.83); (**f**) external validation ROC curves with MRI image data (AUC = 0.89).

The external validation set employed data from ovarian cancer (OC) for verification, taking into consideration both the radiologist’s assessment recommendations for lymph node metastasis and OC’s MRI images. The experimental results on the external validation set indicated that the performance of the model was further validated and confirmed. Without MRI image data, the model achieved a sensitivity of 88.16%, a specificity of 91.65%, an accuracy of 89.26%, and an AUC (ROC curve) of 0.83. However, when combined with MRI imaging and evaluation data, the sensitivity was approximately 91.14%, the specificity was about 92.16%, the accuracy was around 92.26%, and the AUC (ROC curve) was 0.89, as depicted in Figure 8.

**Figure 9:**
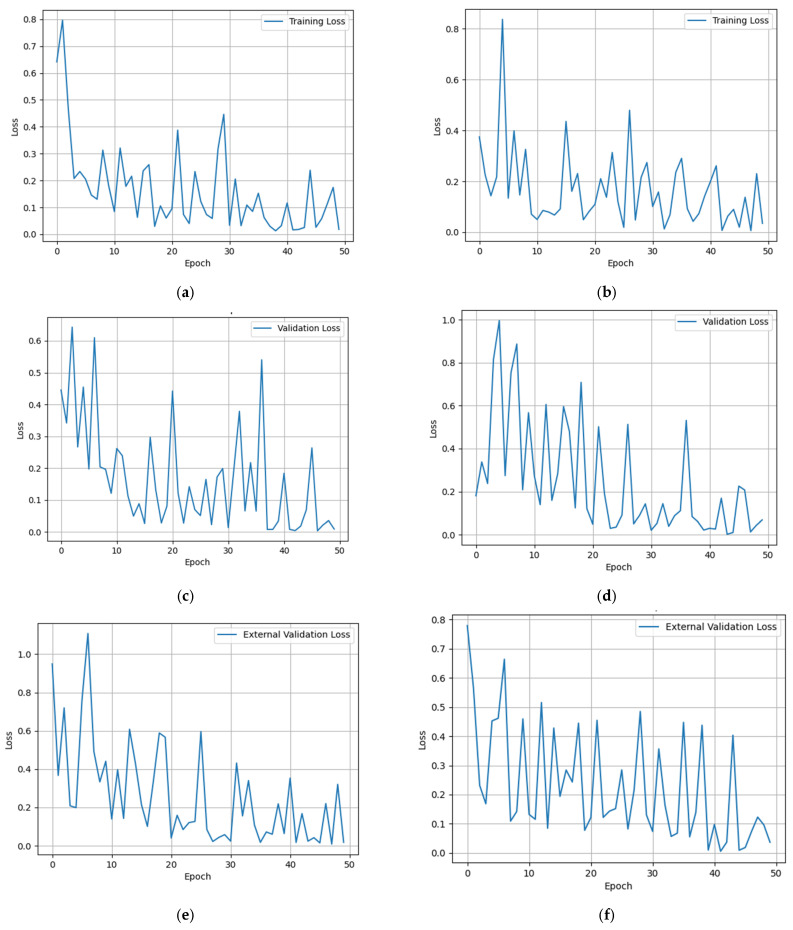
(**a**) Training loss without MRI image data; (**b**) training loss with MRI image data; (**c**) validation loss without MRI image data; (**d**) validation loss with MRI image data; (**e**) external validation loss without MRI image data; (**f**) external validation loss with MRI image data.

Furthermore, to provide a more intuitive display of the model’s training process, the study plotted the loss function against the number of training iterations, as illustrated in Figure 9. Overall, as training progressed, the model’s loss value gradually decreased, indicating that the model was converging, and its performance was continuously optimized.

These results indicated that by integrating multimodal-image and non-image data, the federated-learning model performed exceptionally well, even without the presence of image information, and further improved its predictive accuracy upon incorporating MRI images and evaluation data. Similar sensitivity and specificity were observed in the ex-ternal validation set, further confirming the robustness and reliability of the framework.

## 6. Discussion

Gynecological malignant tumors, particularly CC and EC, are well-known for their invasive metastatic properties. In this context, LNM has emerged as a vital prognostic marker, due to its pivotal role in patient recurrence and overall survival rates. As such, employing effective imaging techniques for LNM evaluation is paramount.

Existing imaging technologies present challenges in predicting LNM. While CT and MRI are the most commonly used imaging modalities for lymph node assessment, their diagnostic efficiency is relatively low. Compared to PET/CT, CT and MRI offer lower sensitivity but higher specificity. Previous studies showed that the sensitivity of CT and MRI ranges from 31% to 58% and 34% to 71%, respectively [14,15,16,17,18]. Furthermore, interpreting CT and MRI images to determine the occurrence of LNM requires seasoned radiologists. Although PET/CT has demonstrated an exceptional diagnostic efficiency of 80.15%, detecting metastatic lesions smaller than 5 mm remains a challenge [20].

Against this backdrop, this study introduced an assessment framework based on multi-modal federated learning. The method innovatively integrated non-imaging clinical data (radiologists’ evaluations of CT, MRI, and PET/CT) with MRI image-assessment results. This fusion strategy, supported by federated learning, not only bolstered the predictive capability for LNM, but also redefined the sharing and processing methods for multi-center data. The approach sought to harmonize prediction standards across multiple centers, potentially elevating diagnostic standards in local hospitals and mitigating diagnostic discrepancies between medical centers. Clinically, this method can be instrumental in guiding oncologists and surgeons in making informed therapeutic decisions based on a more comprehensive and accurate assessment of the presence of LNM. By providing a more holistic view of a patient’s condition, it ensures that treatment plans are tailored to the individual’s specific needs, enhancing patient outcomes. In essence, it offers robust support for standardizing and enhancing the precision of malignant gynecological tumor evaluations, fostering more consistent and accurate therapeutic decision-making.

In the realm of LNM evaluation, the federated-learning model showcased marked superiority over established imaging techniques. Even without the inclusion of MRI data, the model delivered a sensitivity of 92.30%, a specificity of 92.15%, and an accuracy of 92.17%. In contrast, CT reported a sensitivity and a specificity of 32.63% and 92.15% respectively, and MRI revealed figures of 35.9% and 93.37%, respectively. Interestingly, even when the federated-learning model was compared with PET/CT—which had a sensitivity of 57.69% and a specificity of 85.71%—the model, upon integrating MRI data, exhibited enhanced metrics: i.e., a sensitivity of 94.12%, a specificity of 96.69%, and an accuracy of 95.16%. The external validation set further reaffirmed the model’s robustness and reliability, underlining its distinct advantage in LNM evaluation. The findings not only underscore the model’s prowess in comparison to traditional approaches, but also position it as a promising paradigm shift in advancing LNM assessments.

Previous scholars also provided valuable exploration and backing in this domain. Lanhong et al. [32] attempted to consolidate imaging data to enhance LNM diagnostic accuracy. Although they recognized the significance of multi-modal combinations, their research still faced challenges in integrating non-imaging data and sharing data across hospitals. The study of Silva et al. [33], despite enhancing the model’s generalizability through federated learning for inter-hospital data sharing, remained limited to a single imaging modality and did not integrate non-imaging data. Liu et al. [34] discussed the limitations of single-modal machine learning methods in predicting LNM, emphasizing the importance of multi-modal and multi-center data, and suggested future research directions.

As this study delved deeply into the complexities of predicting LNM in gynecological malignant tumors, the endeavors of other researchers in similar or adjacent fields offer valuable insights. A study focused on muscle-atrophy assessment in head and neck cancer patients [35] showcased how advanced deep-learning techniques, specifically CNN and LSTM, can be harnessed for precise medical-image analysis. The MLNet, a deep learning network based on meta-heuristic algorithms [36], presented a fresh perspective for automated CC diagnosis. By integrating various algorithms or models, more effective and precise prediction methods might emerge.

Additionally, some researchers explored the value of transfer learning in automated CC tumor segmentation [37], successfully applying it to the diffusion-weighted magnetic resonance imaging of uterine malignant tumors. Liu et al. [34] presented a fresh perspective on machine learning in MRI-image diagnosis of prostate cancer. These innovative approaches provide novel viewpoints on how to apply existing knowledge to new areas.

For the stratification of recurrence risk in locally advanced CC patients, some researchers successfully employed multi-modal transformer networks, integrating different modalities of medical-image data, such as CT and MRI, offering more comprehensive and accurate risk assessment methods [38].

Finally, regarding the prediction of LNM in CC patients, some researchers used digital pathological features extracted from biopsy slides [30]. They successfully predicted LNM based on the vision transformer (ViT) [30] and recursive neural network (RNN) framework, and the model’s performance was validated through external testing and prospective datasets. Compared to the method of this study, this approach offered a contrast, showcasing the value of multi-modal data in different research contexts.

While this study achieved noteworthy advancements, several limitations warrant attention. First, due to the limited sample sizes and the study’s restriction to a single center, the model’s generalizability and statistical power might be compromised. Second, the current research primarily relied on MRI for image assessment, somewhat restricting the model’s versatility and adaptability and indicating a need to integrate diverse imaging techniques in the future.

Regarding future endeavors, several clear directions have been settled, all building on the foundation of our current work. A primary strategy is to increase sample quantities and varieties, including more imaging sequences, clinical data, laboratory data, and gynecological examination records, with the aim of constructing a more comprehensive and accurate LNM-prediction model. With the deepening advancement of AI in diagnostic technology, our multi-modal federated-learning framework is poised to serve as a guidepost in the field, directing researchers toward new research directions and innovative ideas. Additionally, we plan to integrate advanced algorithms, such as the ViT, and combine them with knowledge graphs and clinical expertise to further boost the model’s predictive performance. Concurrently, considering the model’s broad applicability, we are exploring the use of techniques such as transfer learning to predict LNM in other types of cancers.

As an ongoing research endeavor, more data types will be incorporated into the multi-modal federated-learning model and it will be validated across various centers.

## 7. Conclusions

In the complex field of gynecological malignancies, precise early detection of LNM in CC and EC patients is crucial. A novel multi-modal federated-learning framework was employed to address this detection, integrating non-imaging clinical data with MRI images and overcoming the limitations of traditional imaging techniques. Utilizing a sophisticated neural network, the methodology seamlessly combined MRI and clinical data, enhancing prediction accuracy and data confidentiality.

In conclusion, this research signifies more than progress in diagnosing gynecological malignancies. It also highlights the transformative potential of uniting various data types within a federated structure, guiding future research, with a focus on diagnostic accuracy and patient welfare.

## Figures and Tables

**Table 1:** Characteristics of the patients with cervical or endometrial carcinoma.

	N (%)
Median age (range)	49 (19–78)
Cervical carcinoma	423
Endometrial carcinoma	144
Lymph node metastasis	
Cervical carcinoma	
No	334(78.96)
Yes	89 (21.04)
Endometrial carcinoma	
No	130(90.28)
Yes	14(9.72)
FIGO 2009 stage	
Cervical carcinoma	
IA1	8 (1.89)
IA2	18 (4.26)
IB1	219 (51.77)
IB2	33 (7.80)
IIA1	61 (14.42)
IIA2	84 (19.86)
Endometrial carcinoma	
IA	102 (70.83)
IB	18 (12.5)
II	6 (4.17)
IIIA	4 (2.78)
IIIC1	9 (6.25)
IIIC2	5 (3.47)
LVSI	
Cervical carcinoma	
No	310 (73.29)
Yes	113 (26.71)
Endometrial carcinoma	
No	123 (85.42)
Yes	21 (14.58)
Stromal invasion	
Cervical carcinoma	
<1/3	171 (40.43)
1/3–2/3	86 (20.33)
>2/3	166 (39.24)
Endometrial carcinoma	
<1/2	117 (81.25)
>1/2	27 (18.75)
Histology	
Cervical carcinoma	
Squamous cell carcinoma	321 (75.89)
Adenocarcinoma	81 (19.15)
Adenosquamous cell carcinoma	8 (1.89)
Neuroendocrine carcinoma	9 (2.13)
Clear cell carcinoma	1 (0.24)
Rhabdomyosarcoma	1 (0.24)
Carcinosarcoma	1 (0.24)
Genital wart-like carcinoma	1 (0.24)
Endometrial carcinoma	
Endometrioid carcinoma	131 (90.97)
Clear cell carcinoma	6 (4.17)
Serous carcinoma	4 (2.78)
Carcinosarcoma	3 (2.08)
Grade	
Cervical carcinoma	
1	10 (2.36)
2	197 (46.57)
3	70 (16.55)
Non-keratinizing SCC	26 (6.15)
Keratinizing SCC	5 (1.18)
Not reported	91 (21.75)
Endometrial carcinoma	
1	33 (22.92)
2	64 (44.44)
3	26 (18.06)
Not reported	8 (5.56)

SCC, squamous cell carcinoma; FIGO, International Federation of Gynecology and Obstetrics; LVSI, lymphovascular space involvement.

**Table 2:** Medical data overview.

Field	Meaning
Hospital ID	Records the unique identifier of the patient within the hospital
Diagnosis result	Indicates the patient’s disease diagnosis, including cervical and endometrial malignant tumors
Preoperative CT	Records the results of CT evaluation conducted before surgery
MRI	Records the results of MRI evaluation conducted before surgery
PET/CT LNM	Indicates the LNM in the pelvic and abdominal cavity evaluated by PET/CT
PET results	Record the results of PET evaluation conducted before surgery
CT LNM	Indicates the LNM in the pelvic and abdominal cavity evaluated by CT
CT results	Record the results of CT evaluation conducted before surgery

**Table 3:** Distribution of samples and data splitting among clients.

Client	Positive Samples	Negative Samples	Training Set	Testing Set	Validation Set
Client 0	111	115	181 (8:2)	45 (8:2)	341 (Client 1)
Client 1	226	115	273 (8:2)	68 (8:2)	226 (Client 0)

**Table 4:** Separate and combined use of computed tomography, magnetic resonance imaging, and positron emission tomography-computed tomography for the assessment of lymph node metastasis.

	Pathology		Total
	Positive for LNM	Negative for LNM	
CT			439
Positive for LNM	31	27	
Negative for LNM	64	317	
MRI			440
Positive for LNM	28	24	
Negative for LNM	50	338	
PET/CT			393
Positive for LNM	45	45	
Negative for LNM	33	270	
CT + MRI			336
Positive for LNM	27	25	
Negative for LNM	45	239	
CT + PET/CT			308
Positive for LNM	42	49	
Negative for LNM	29	188	
MRI + PET/CT			292
Positive for LNM	33	43	
Negative for LNM	27	189	
CT + MRI + PET/CT			230
Positive for LNM	31	40	
Negative for LNM	24	135	

LNM, lymph node metastasis; CT, computed tomography; MRI, magnetic resonance imaging; PET/CT positron emission tomography–computed tomography.

**Table 5:** Comparison of the diagnostic efficiency of the separate and combined use of computed tomography, magnetic resonance imaging, and positron emission tomography–computed tomography for lymph node metastasis.

	Group	Sensitivity	Specificity	PPV	NPV	Accuracy	AUC
Efficiency	CT	32.63%	92.15%	53.45%	83.20%	79.27%	0.624 (0.555–0.693)
	MRI	35.9%	93.37%	53.85%	87.11%	83.18%	0.646 (0.571–0.721)
	PET/CT	57.69%	85.71%	50.0%	89.11%	80.15%	0.717 (0.647–0.787)
	C-M	37.5%	90.53%	51.92%	84.15%	79.17%	0.640 (0.561–0.719)
	C-P	59.15%	79.32%	46.15%	86.64%	74.68%	0.692 (0.618–0.767)
	M-P	55.0%	81.47%	43.42%	87.1%	76.03%	0.682 (0.601–0.764)
	C-M-P	56.36%	77.14%	43.06%	85.44%	72.17%	0.668 (0.582–0.753)
*p* value	CT vs. MRI	0.652	0.532	0.967	0.127	0.138	0.5528
	CT vs. PET/CT	0.001 *	0.008 *	0.682	0.028 *	0.752	0.0172 *
	MRI vs. PET/CT	0.006 *	0.001 *	0.659	0.423	0.258	0.0846
	C-M vs. C-P	0.01 *	<0.001 *	0.507	0.438	0.176	0.2359
	C-M vs. M-P	0.044 *	0.003 *	0.344	0.291	0.346	0.3595
	C-M vs. C-M-P	0.034 *	<0.001 *	0.365	0.834	0.055	0.5679
	C-P vs. M-P	0.632	0.559	0.724	0.789	0.701	0.8318
	C-P vs. C-M-P	0.753	0.595	0.752	0.634	0.515	0.6139
	M-P vs. C-M-P	0.883	0.284	0.977	0.469	0.317	0.7719
	CT vs. C-P	0.001 *	<0.001 *	0.385	0.265	0.139	0.0942
	PET/CT vs. C-P	0.856	0.048 *	0.605	0.391	0.084	0.5746
	MRI vs. M-P	0.025 *	<0.001 *	0.246	0.891	0.017 *	0.4212

*: *p* < 0.05. PPV, positive predictive value; NPV, negative predictive value; AUC, area under the curve; CT, computed tomography; MRI, magnetic resonance imaging; PET/CT positron emission tomography–computed tomography. C-M: CT and MRI; C-P: CT and PET/CT; M-P: MRI and PET/CT; C-M-P: CT, MRI and PET/CT.

## Data Availability

The data that support the findings of this study are available upon reasonable request from the corresponding author.
